# Interleukin-2 expands neuroprotective regulatory T cells in Parkinson’s disease

**DOI:** 10.1515/nipt-2022-0001

**Published:** 2022-06-21

**Authors:** Milica Markovic, Pravin Yeapuri, Krista L. Namminga, Yaman Lu, Maamoon Saleh, Katherine E. Olson, Howard E. Gendelman, R. Lee Mosley

**Affiliations:** Department of Pharmacology and Experimental Neuroscience, Center for Neurodegenerative Disorders, University of Nebraska Medical Center, 68198 Omaha, NE, USA.; Department of Pharmaceutical Sciences, College of Pharmacy, University of Nebraska Medical Center, Omaha, NE, USA

**Keywords:** immune tolerance, interleukin-2, neuroprotection, Parkinson’s disease, regulatory T cells, Tregs

## Abstract

**Background:**

Pharmacological approaches that boost neuroprotective regulatory T cell (Treg) number and function lead to neuroprotective activities in neurodegenerative disorders.

**Objectives:**

We investigated whether low-dose interleukin 2 (IL-2) expands Treg populations and protects nigrostriatal dopaminergic neurons in a model of Parkinson’s disease (PD).

**Methods:**

IL-2 at 2.5 × 10^4^ IU/dose/mouse was administered for 5 days. Lymphocytes were isolated and phenotype determined by flow cytometric analyses. To 1-methyl-4-phenyl-1,2,3,6-tetrahydropyridine (MPTP) intoxicated mice, 0.5 × 10^6^ of enriched IL-2-induced Tregs were adoptively transferred to assess the effects on nigrostriatal neuron survival.

**Results:**

IL-2 increased frequencies of CD4^+^CD25^+^CD127^low^FoxP3^+^ Tregs that express ICOS and CD39 in blood and spleen. Adoptive transfer of IL-2-induced Tregs to MPTP-treated recipients increased tyrosine hydroxylase (TH)^+^ nigral dopaminergic neuronal bodies by 51% and TH^+^ striatal termini by 52% compared to control MPTP-treated animal controls.

**Conclusions:**

IL-2 expands numbers of neuroprotective Tregs providing a vehicle for neuroprotection of nigrostriatal dopaminergic neurons in a pre-clinical PD model.

## Introduction

Parkinson’s disease is the most common neurodegenerative motor disorder [1]. The characteristic phenotype of the disorder includes tremors at rest and bradykinesia that result from loss of the neurotransmitter dopamine, which is due, in large part, to the loss of dopamine-synthesizing neurons that originate in the substantia nigra (SN) pars compacta and innervate to the striatum. As one of many synucleinopathies, intraneuronal inclusions of misfolded and aggregated α-synuclein (α-syn) and ubiquitin accumulate to form Lewy bodies, which are hallmarks of PD and considered posthumously diagnostic for PD [2, 3]. A second characteristic hallmark of PD is chronic inflammation mediated by innate immune cells, such as microglia and infiltrating macrophages. Abundant evidence indicates that high levels of inflammation lead to an increased oxidative state in PD and play a major role in disease progression and possibly etiology [4–6]. Activated microglia secrete neurotoxic mediators and sufficiently increase oxidative stress to induce misfolding and modification of α-syn, which is secreted or released into the extraneuronal environment upon cell injury or death, and activates surrounding microglia to perpetuate a chronic inflammatory state. Moreover, inflammatory molecules secreted by microglia also upregulate expression of monomeric α-syn in neurons and promote its aggregation [7]. Activated microglia can also elevate astrocytes to a reactive A1 neurotoxic phenotype and play an important role in persistent chronic neuroinflammation and α-syn aggregate formation [8–12]. Notably, neuronal exosomes that contain monomeric and oligomeric α-syn are associated with spreading pathogenic α-syn species to other neurons and glia that, in turn, support persistent inflammatory states [13–18]. In contrast, immune forces that control and regulate chronic inflammation are diminished in PD [6, 19], [20], [21], [22]. Indeed, several reports show that regulatory T cells (Tregs), which maintain active immunological tolerance and attenuate inflammatory responses, are significantly diminished in PD patients, while activated pro-inflammatory myeloid and effector T cells (Teffs) are increased [21, 23], [24], [25].

Based on extensive pre-clinical studies and clinical trials, we and others identified that Tregs can transform pro-inflammatory, neurotoxic environments to more anti-inflammatory and neurotrophic environments, wherein dopaminergic neurons are rescued or spared from a degenerative fate [20, 23, 24, 26], [27], [28], [29]. Indeed, immunomodulatory agents such as granulocyte-macrophage colony-stimulating factor (GM-CSF) increase Treg numbers and activity with parallel neuroprotective activities in PD and Alzheimer’s disease (AD) [20, 23], [24], [25, 27], [28], [29], [30], [31]. Many regulatory mechanisms of induced Tregs (iTregs) utilize known pathways for improving disease outcomes; however, whether all pathways are operative in PD have yet to be determined. Therefore, to compensate Treg deficits, optimal levels of iTregs or iTreg activity could better control disease-initiating inflammatory activities and potentially mitigate disease progression. Thus, novel therapeutic approaches would utilize immunomodulatory agents to induce Tregs to restore diminished numbers and/or activity, attenuate neuroinflammation, and re-establish or maintain immune tolerance with intervention of disease progression in PD patients. Indeed, while GM-CSF seems to satisfy those directives, the GM-CSF signaling pathway that induces Tregs seems not to be one of direct Treg interaction [27, 32].

The rationale for utilizing interleukin-2 (IL-2) rests in its essential defined roles and direct interaction in survival and expansion of new or existing Tregs. IL-2 upregulates forkhead box P3 (FoxP3) expression during Treg development in the thymus and is involved in Treg differentiation, lineage stability, proliferation, and function [33]. Tregs constitutively express the high-affinity receptor for IL-2, while its expression by other subsets of T cells is induced after activation [34]. IL-2 signaling is also a key component by which CD4^+^CD25^+^FoxP3^+^ iTregs exhibit functional dominance and can outgrow other T-cell types that typically express lower levels of the IL-2 receptor α chain (CD25) [35–38]. Therefore, in the steady state, functional iTregs respond better to IL-2 than other T cells and do so in a direct manner, rather than via intermediary cell interactions [39]. This may account for the ability of low doses of IL-2 to preferentially increase Treg numbers without global immune activation of other T cell types [40–42]. However, whether IL-2 selectively induces new Tregs or merely expands existing, but possibly deficient, Tregs is currently unknown and untested in PD. Therapies such as low-dose IL-2 or IL-2/anti-IL-2 antibody complexes have been advanced in the clinic to preferentially expand Treg populations as a treatment for chronic inflammatory autoimmune diseases [43]. Some neuroprotective effects of low-dose IL-2 treatment have been found in AD mice [44] and in amyotrophic lateral sclerosis (ALS) patients [45]. Nevertheless, no study thus far has investigated the effects of low-dose IL-2 in PD or pre-clinical PD models.

Herein, we sought to elucidate the impact of low-dose IL-2-mediated transformation and expansion of Tregs in the peripheral blood and lymphoid tissues of mice, and to evaluate the neuroprotective capabilities of Tregs generated by this pharmacological intervention in a PD animal model.

## Materials and methods

### Animals and IL-2 treatment

C57BL/6 male mice (5–6 weeks old) were purchased from Jackson Laboratories (Bar Harbor, ME). Animals were housed and maintained in accordance with the National Institutes of Health institutional guidelines and approved by the Institutional Animal Care and Use Committee (IACUC) of the University of Nebraska Medical Center. Following a period of acclimation, mice were injected intraperitoneally (i.p.) with daily, low-dose (2.5 × 10^4^ IU) human recombinant interleukin-2 (PeproTech, Cranbury, NJ, USA) [40–42] for 5 days. For neuroprotection experiments, mice were injected with either vehicle (Dulbecco’s phosphate-buffered saline, DPBS) (Sigma-Aldrich, St. Louis, MO, USA) at 10 mL/kg body weight or 1-methyl-4-phenyl-1,2,3,6-tetrahydropyridine hydrochloride (MPTP-HCl) in DPBS (Sigma-Aldrich). Mice received 4 subcutaneous (s.c.) injections of MPTP-HCl (16 mg/kg of MPTP free base) or DPBS, each injection at 2 h intervals. Precautions for handling MPTP were according to the MPTP safety and handling protocol [46]. On day 7 post-MPTP intoxication, mice were sacrificed, and brains were harvested and processed for assessment of neuronal survival.

### Flow cytometric analysis

**Figure 1:**
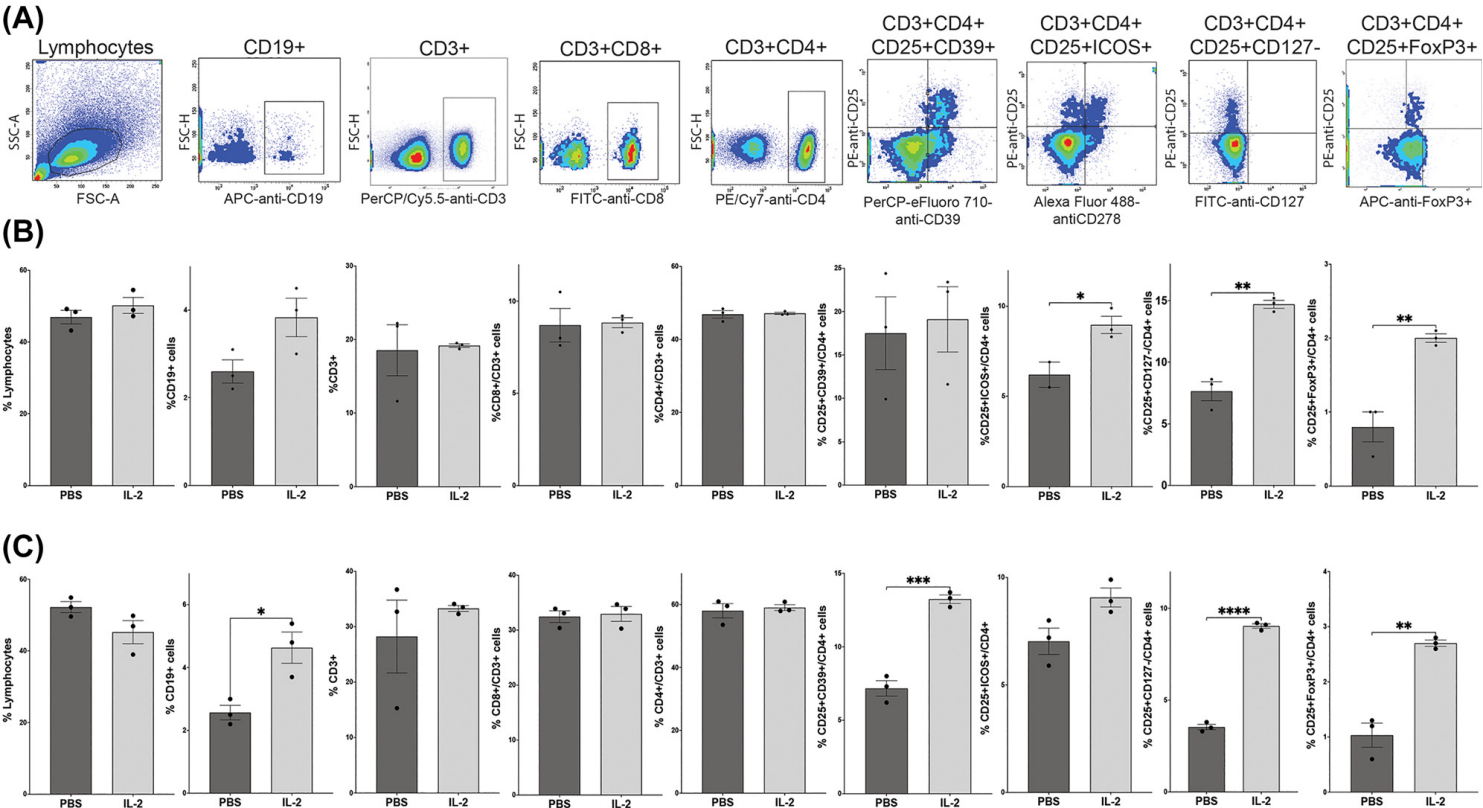
(A) Representative gating strategy for flow cytometric analysis of lymphocyte population in the whole blood of mice treated with PBS or 2.5 × 10^4^ IU rIL-2. Quantification of CD3^+^, CD19^+^, CD4^+^, CD8^+^, CD4^+^CD25^+^CD127^low^, CD4^+^CD25^+^CD39^+^, CD4^+^CD25^+^ICOS^+^, and CD4^+^CD25^+^FoxP3^+^ cells in (B) whole blood and (C) spleen of treated mice. Differences in mean ± SEM (*n*=3 mice per group) between groups were determined significant when p<0.05.

Following 5 days of treatment with low-dose IL-2 or DPBS, whole blood and spleens were collected to determine T- and B-cell profiles via flow cytometric (FCM) analysis. Peripheral blood and splenocytes were fluorescently labeled using antibodies against cell surface antigens including CD19, CD3, CD4, CD8, CD25, CD39, ICOS, and CD127, and for the intracellular marker, FoxP3. Whole blood (50 μL) and splenocytes (1 × 10^6^) were labeled with PerCP Cyanine 5.5-anti-CD3 (eBioscience/Thermo Fisher Scientific, Walltham, MA, USA), FITC-anti-CD8 (eBiosciences), APC-anti-CD19 (eBiosciences), PE-anti-CD25 (eBiosciences), PE-cyanine7-anti-CD4 (eBiosciences), PerCP-eFluor710-anti-CD39 (eBiosciences), Alexa Fluor488-anti-CD278 (ICOS) (Biolegend, San Diego, CA, USA), and FITC-anti-CD127 (Biolegend). Isotype-matched antibodies and fluorescence minus one (FMOs) served as negative controls. Intracellular staining was performed following permeabilization for 45 min at 4 °C using FoxP3/Transcription Factor Staining Buffer Set (eBioscience). Following fixation, cells were labeled with APC-anti-FoxP3 (eBioscience). Samples were analyzed with an LSRII flow cytometer and FACSDiva Software (BD Biosciences, San Diego, CA, USA), and all cell frequencies were gated from the total lymphocyte population (Figure 1A).

### Treg isolation and adoptive transfer

For mouse studies, CD4^+^CD25^+^ T cells were isolated from spleens and lymph nodes using EasySep Mouse CD4^+^CD25^+^ Regulatory T Cell Isolation Kit II (StemCell, Cambridge, MA, USA) per the manufacturer’s instructions. CD4^+^CD25^+^ Tregs were >85% enriched as determined by FCM analysis. To MPTP-intoxicated recipient mice, 0.5 × 10^6^ CD4^+^CD25^+^ enriched Tregs were adoptively transferred via tail vein injection 8–12 h after the final MPTP treatment; an interval which allows clearance of extracellular MPTP and MPP+ [4, 20, 27, 31, 46, 47].

### Perfusion and immunohistochemistry

Seven days after MPTP intoxication, mice were terminally anesthetized with pentobarbital (Fatal Plus, Vortech Pharmaceutical, Dearborn, MI, USA) and transcardially perfused with PBS followed by 4% paraformaldehyde (PFA) (Sigma-Aldrich) in PBS. Brains were harvested after perfusion and processed to assess survival of dopaminergic neuronal cell bodies in the substantia nigra (SN) and termini in the striatum. To evaluate neuronal bodies, cryopreserved midbrains were sectioned at 30 μm and immunostained for tyrosine hydroxylase (TH) (anti-TH, 1:2000, EMD Millipore, Burlington, MA, USA) and counterstained for Nissl substance [27, 47]. To assess dopaminergic termini, 30 μm striatal sections were labeled with anti-TH (1:1,000, EMD Millipore). To visualize antibody-labeled tissues, sections were incubated in streptavidin-HRP solution (ABC Elite Vector Kit, Vector Laboratories, Burlingame, CA USA) and color was developed using an H_2_O_2_ generation system in the presence of diaminobenzidine (DAB) chromogen (Sigma-Aldrich). Estimated neuron numbers were quantified by a blinded investigator and unbiased stereological analysis using StereoInvestigator software (MBF Bioscience, Williston, VT, USA) [27, 47]. Density of dopaminergic neuron termini in the striatum was determined by digital densitometry using Image J software (National Institutes of Health, Bethesda, MD, USA).

### Statistical analysis

All values are expressed as mean ± SEM. Differences in between-group means for neuronal counts were analyzed using ANOVA followed by Newman–Keuls post hoc test. For differences between groups in populations of lymphocytes Student’s t-test was used (mean ± SEM, *n*=3). For all studies, data were analyzed using GraphPad Prism 9.3.1 software (La Jolla, CA, USA).

## Results

Treatment of mice with low-dose IL-2 produced 2- to 3-fold higher percentages of CD4^+^CD25^+^ Tregs in mice regardless of CD127^low^ or FoxP3^+^ Treg phenotype as demonstrated by flow cytometric analysis and compared to PBS-treated controls (Figure 1A–C). In both blood and spleen of IL-2-treated mice, frequencies of CD4^+^CD25^+^CD127^low^ Tregs were significantly elevated by at least 2-fold, while CD4^+^CD25^+^FoxP3^+^ Tregs were 3-fold higher than those of PBS controls. Percentages of CD4^+^CD25^+^ Tregs that express CD39 or ICOS cell surface markers were also elevated in spleens of mice treated with IL-2 compared to PBS-treated mice. CD4^+^CD25^+^CD39^+^ Treg frequencies were double in the spleen, but unchanged in the blood, whereas CD4^+^CD25^+^ICOS^+^ Tregs were significantly elevated in blood and somewhat higher in the spleen compared to PBS control, though not significantly. Additionally, IL-2 treatment elevated the frequencies of CD4+CD25+FoxP3+CD39+ Tregs in blood and spleen compared to controls from 0.5% ± 0.2 to 1.7% ± 0.8 and 0.03% ± 0.03 to 1.1% ± 0.9%, respectively, though those increases were not statistically significant. Percentages of CD19^+^ B lymphocytes in peripheral blood of IL-2 treated mice were elevated, though not significantly (Figure 1B), whereas those in spleen were significantly increased (Figure 1C). Other markers for T-lymphocyte populations showed no significant differences in expression from IL-2-treated mice compared to PBS controls.

**Figure 2:**
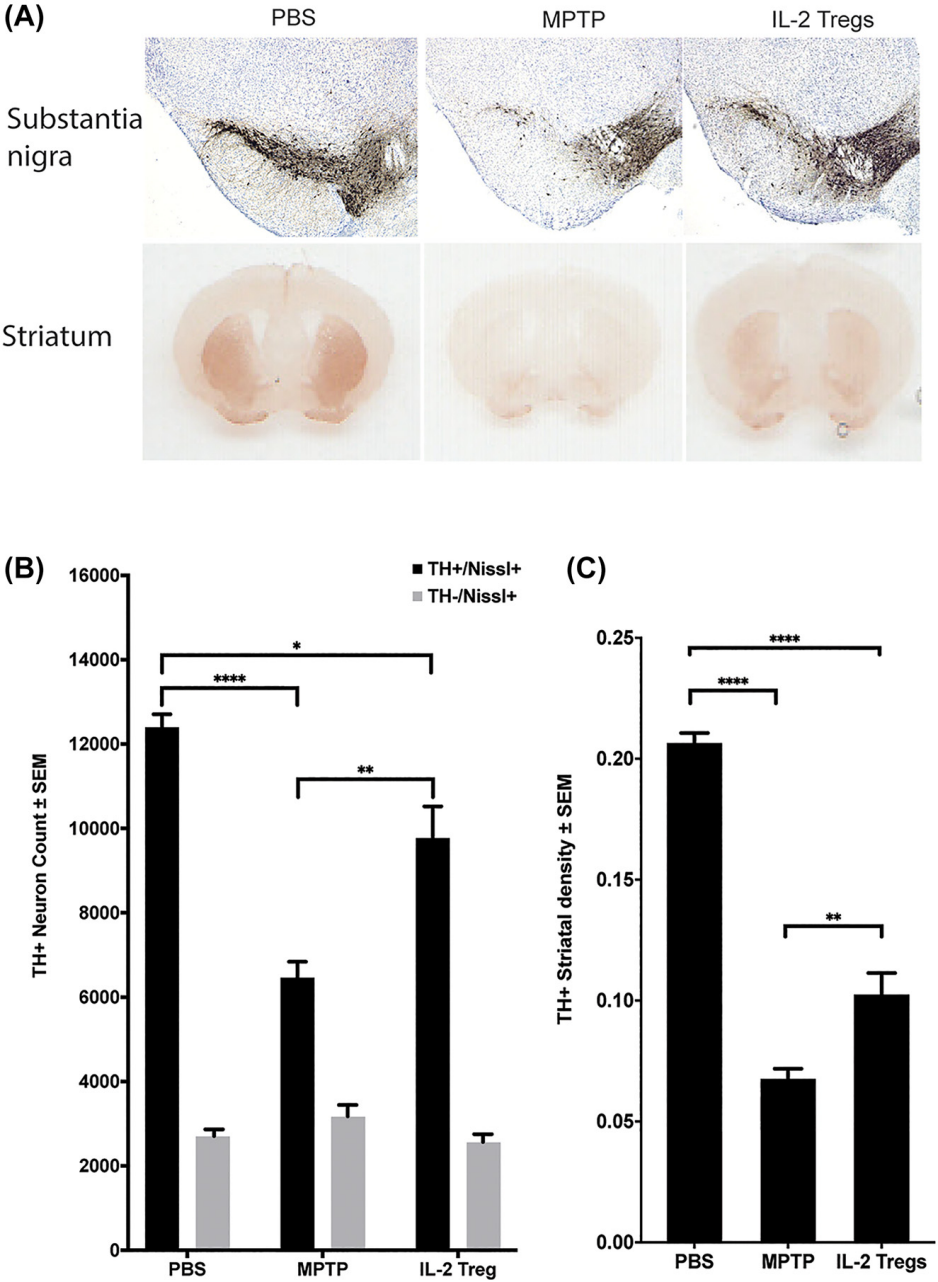
Neuroprotection following adoptive transfer of low-dose IL-2-induced Tregs. (A) Representative images of TH^+^/Nissl^+^ dopaminergic neurons within the substantia nigra (SN, upper row) and projections into the striatum (STR, bottom row) of recipient mice treated with PBS (*n*=7), MPTP (n=6), or MPTP followed by adoptive transfer of Tregs from donor mice treated with 2.5 × 10^4^ IU of IL-2 (IL-2 Treg) (n=6). (B) Quantification of total numbers of surviving dopaminergic (TH^+^/Nissl^+^) and non-dopaminergic (TH^−^/Nissl^+^) neurons within the SN following MPTP intoxication and adoptive transfer of 0.5 × 10^6^ Tregs. (C) Densitometry analysis of TH^+^ termini within the STR with MPTP intoxication followed by adoptive transfer of Tregs. Differences in means ± SEM were determined where p<0.05.

To assess the neuroprotective capacity of IL-2-induced Tregs, lymph node and splenic T cells were isolated from low-dose IL-2 treated donors, enriched for CD4^+^CD25^+^ Tregs, and adoptively transferred to MPTP-treated recipient mice. Seven days after adoptive transfer of Tregs, ventral midbrains and striata of recipient mice were sectioned, processed, stained for TH expression, and assessed for TH+ dopaminergic neuron survival (Figure 2A). Adoptive transfer of Tregs from donor mice treated with low-dose IL-2 increased TH^+^/Nissl^+^ neuron survival 51% to 9773 ± 750 compared to MPTP intoxication alone (6460 ± 380) (Figure 2B). Likewise, densitometric analysis of TH^+^ striatal termini demonstrated a significant neuroprotective effect following adoptive transfer of IL-2-induced Tregs by rescuing 52% more dopaminergic striatal termini compared to MPTP-treated mice (Figure 2C).

## Discussion

Tregs are a subset of CD4^+^ T cells that play a major role in the initial establishment and maintenance of immune tolerance and sustaining immune homeostasis. Functional mechanisms by which Tregs function include disrupting antigen presentation and co-stimulation by antigen presenting cells (APCs), suppressing proliferative activities of CD4^+^ and CD8^+^ Teffs, and attenuating pro-inflammatory functions of myeloid lineage cells, such as microglia [48]. Tregs are initially derived from the thymus (natural or nTregs), but also arise from peripheral naïve T cells or are transformed from Teffs in the periphery (induced Tregs or iTregs); the latter mainly develop from activated CD4^+^ T cells in the presence of TGF-β and IL-2. Tregs are broadly identified as CD3^+^CD4^+^CD25^+^FoxP3^+^ cells in both mice and humans. In addition, an inverse correlation of IL-7 receptor (CD127) expression and FoxP3 expression exist, whereby Tregs are characterized as low CD127-expressing cells [49]. Here, we demonstrate that treatment with low doses of IL-2 induces a treatment-specific Treg population with overexpression of immunosuppressive Treg cell markers. Our data show a distinct increase in representation of CD3^+^CD4^+^CD25^+^CD127^low^ Tregs that express FoxP3 in the blood and spleen of IL-2 treated animals compared to PBS-treated controls. Frequencies of Tregs were also increased that express CD39, which together with CD73, comprise two surface expressed ectonucleotidases that catalyze the degradation of ATP to adenosine, which acts on adenosine A2a receptors to inhibit dendritic cell (DC) presentation of antigens and suppress proliferation of activated Teffs [50]. In the spleens of IL-2-treated mice, increased numbers of CD4^+^CD25^+^CD39^+^ Tregs were double those of PBS controls. ICOS signaling in Tregs is also important for generation, proliferation, survival, and suppressive activities of Tregs [51]. ICOS^+^ Tregs exhibit superior inhibitory capacity that is partly attributable to ICOS-mediated induction of IL-10, an anti-inflammatory cytokine, which with ICOS-signaling can synergize to broadly participate in anti-inflammatory control of responses [52, 53]. Indeed, in these studies, peripheral blood and spleen CD25^+^ICOS^+^ Treg frequencies were also increased in IL-2-treated mice.

Next, we elucidated whether Treg populations from IL-2-treated donor mice have the capacity to affect dopaminergic neurodegeneration and spare dopamine neuronal bodies in the substantia nigra and termini in the striatum of MPTP-intoxicated mice. We previously demonstrated that adoptive transfer of natural or pharmacologically-induced [GM-CSF or vasoactive intestinal peptide (VIP)] donor Tregs at doses as low as 1 × 10^6^ CD4^+^CD25^+^ cells/recipient provided significant neuroprotection in the MPTP model [20, 27]. In this study, we adoptively transferred 0.5 × 10^6^ CD4^+^CD25^+^ Tregs that were induced by low doses of IL-2. With 50% fewer Tregs than previous studies, IL-2-induced Tregs spared 51% of nigral dopaminergic neuronal bodies and 52% of the striatal termini in MPTP intoxicated mice. The potency of the neuroprotective effect afforded by low-dose IL-2-induced Tregs in MPTP-recipients might in part be due to increased frequency of Tregs with upregulated ICOS and CD39, which have been shown to define a more functional subset of Tregs that exhibit higher immunsuppressive capacity [53, 54], thus providing greater neuroprotection. Further studies are needed to investigate the dose-dependency of IL-2 induction on Treg populations. With additional immune phenotyping and expression profiles of IL-2-induced Tregs, the mechanism of how these Tregs differ from Tregs induced by other immunomodulatory agents, such as GM-CSF or VIP may be revealed [26], [27], [28, 31]. Therefore, this interventional strategy may lead to sustained suppressive immune response in PD and many other neurodegenerative diseases and autoimmune disorders.
